# Regulation of Myosin Light-Chain Phosphatase Activity to Generate Airway Smooth Muscle Hypercontractility

**DOI:** 10.3389/fphys.2020.00701

**Published:** 2020-06-26

**Authors:** Mayra D. Álvarez-Santos, Marisol Álvarez-González, Samuel Estrada-Soto, Blanca Bazán-Perkins

**Affiliations:** ^1^Biology Area, Facultad de Ciencias, Universidad Nacional Autónoma de México, Mexico City, Mexico; ^2^Laboratorio de Inmunofarmacología, Instituto Nacional de Enfermedades Respiratorias “Ismael Cosío Villegas”, Mexico City, Mexico; ^3^Facultad de Farmacia, Universidad Autónoma del Estado de Morelos, Cuernavaca, Mexico; ^4^Tecnológico de Monterrey, Escuela de Medicina y Ciencias de la Salud, Monterrey, Mexico

**Keywords:** kinase network in contraction, phosphatase subunit 1c, myosin phosphatase target subunit 1, contraction, myosin phosphatase

## Abstract

Smooth muscle is a central structure involved in the regulation of airway tone. In addition, it plays an important role in the development of some pathologies generated by alterations in contraction, such as hypercontractility and the airway hyperresponsiveness observed in asthma. The molecular processes associated with smooth muscle contraction are centered around myosin light chain (MLC) phosphorylation, which is controlled by a balance in the activity of myosin light-chain kinase (MLCK) and myosin light-chain phosphatase (MLCP). MLCK activation depends on increasing concentrations of intracellular Ca^2+^, while MLCP activation is independent of Ca^2+^. MLCP contains a phosphatase subunit (PP1c) that is regulated through myosin phosphatase target subunit 1 (MYPT1) and other subunits, such as glycogen-associated regulatory subunit and myosin-binding subunit 85 kDa. Interestingly, MLCP inhibition may contribute to exacerbation of smooth muscle contraction by increasing MLC phosphorylation to induce hypercontractility. Many pathways inhibiting MLCP activity in airway smooth muscle have been proposed and are focused on inhibition of PP1c, inhibitory phosphorylation of MYPT1 and dissociation of the PP1c-MYPT1 complex.

## Introduction

Kinases and phosphatases are key regulatory components of many cellular processes. Mathematical analysis of kinase–phosphatase pathways suggests that kinases have an important role in modulating the signal amplitude, while phosphatases control the duration of responses (Heinrich et al., 2002). In addition, the balance in the activation of kinases or phosphatases is regulated by many proteins (Kiss et al., 2019). An interesting example of this balance can be observed in smooth muscle. The machinery of smooth muscle contraction includes many kinases and phosphatases, among which myosin light-chain kinase (MLCK) and myosin light-chain phosphatase (MLCP) are prominent.

Smooth muscle can be classified into two types according to its contractile properties: tonic and phasic. Although phasic and tonic smooth muscles differ in the time of contraction maintenance, both share the same signaling and regulatory pathways. In particular, airway smooth muscle becomes tonic after birth because it does not generate spontaneous contractions (Wang et al., 2019). The intrinsic tone of airways is produced by baseline airway smooth muscle contraction and is under both humoral and neuronal control. Stimulation of airway smooth muscle with contractile agonists induces a reduction in airway caliber and decreases airflow, resulting in respiratory dysfunction. In some diseases, such as asthma, sensitivity and responsiveness to these agonists increase and produce an excessive reduction in airway caliber. This phenomenon, called airway hyperresponsiveness, has been attributed to airway smooth muscle hypercontractility, i.e., an enhanced velocity and extent of contraction in response to chemical or physical stimulation (Nair et al., 2017; Sakai et al., 2017).

Few studies have addressed the relationship between the role of MLCP and airway smooth muscle hypercontractility and airway hyperresponsiveness. MLCP-associated signaling is an interesting perspective from which to understand the possible dysfunctions in the contractile response of smooth muscle. Therefore, in this mini-review, we attempt to elucidate the possible role of MLCP in the development and regulation of airway smooth muscle contraction to generate hypercontraction.

## Contractile Machinery in Smooth Muscle

To expand knowledge on the regulation of smooth muscle contraction, specific reviews have covered this topic in detail (Vetterkind and Morgan, 2012). In general, the machinery involved in muscle contraction is centered around force generation through the interaction of myosin filaments with actin; in smooth muscle, actin filaments are very abundant and are attached to structures called dense bodies to form hexagonal matrices, in which myosin occupies the spaces surrounding each strand of actin. Myosins are a large superfamily of motor proteins, and type II myosin is found in all muscles. The type II myosin protein comprises 6 subunits, two of which are myosin heavy chains (MHCs) that become entangled to form a tail, separate into necks, and end with heads that have binding sites for actin. The necks contain two pairs of myosin light chains (MLCs), a 17 kDa pair and a 20 kDa pair; the latter is called regulatory MLC or MLC20 (Takeya, 2016).

## Phosphorylation of MLC20, a Key Component in the Generation of Hypercontraction During Muscle Contraction

MLC20 phosphorylation at the serine 19 (Ser^19^) residue allows interaction between actin and myosin filaments, which results in smooth muscle contraction. This phosphorylation occurs after the increase in intracellular Ca^2+^ (Ca^2+^i) levels induced by contractile agonist stimulation. The high Ca^2+^i levels saturate the binding sites on the calmodulin (CaM) protein and allow the formation of a complex between the C-terminal domain of CaM and the N-terminal domain of MLCK, causing its activation and subsequent phosphorylation of MLC20 (Vetterkind and Morgan, 2012). In addition, the phosphorylation level of MLC20 is associated with increases in the force and stiffness of airway smooth muscle (Luo et al., 2019; Sieck et al., 2019). The mechanism by which MLC20 phosphorylation is increased has been attributed to various factors, such as the enhancement of MLCK activity and expression (Léguillette et al., 2009; Ouedraogo and Roux, 2014; Ojiaku et al., 2018), increases in the contractile protein content and cytoskeletal remodeling (Dogan et al., 2017; Sieck et al., 2019) and the inhibition of MLC20 dephosphorylation (Somlyo and Somlyo, 2003; Kelker et al., 2009; Chiba et al., 2010). Inhibition of MLC20 dephosphorylation reflects the decrease in MLCP activity (Ojiaku et al., 2018; Shaifta et al., 2018) and is a thought-provoking perspective that we review to explain the hypercontractility of airway smooth muscle.

## Structure of MLCP

MLCP is a serine (Ser)-threonine (Thr) phosphatase expressed in smooth muscle whose function is independent of Ca^2+^i levels. This phosphatase is characterized as a heterotrimeric holoenzyme comprising a catalytic subunit that functions as a type 1c phosphoprotein phosphatase (PP1c), a regulatory subunit called myosin phosphatase target subunit 1 (MYPT1), and an accessory subunit of 20 kDa called M20 that is linked to MYPT1 (Korrodi-Gregório et al., 2014). In addition to dephosphorylating MLC20, this trimeric holoenzyme exhibits phosphatase activity toward other proteins, such as adducin, Tau, merlin, calcineurin-A, interleukin-16, Rb, moezin, and ezrin, and mediates RhoA signaling (Kiss et al., 2019). The regulatory subunit MYPT1 is central to the spatiotemporal regulation of MLCP because the amino terminus of MYPT1 forms a platform for allosteric interaction with PP1c, and this interaction defines the substrate specificity (Grassie et al., 2011; Korrodi-Gregório et al., 2014).

## Regulation of MLCP

The mechanisms of MLCP regulation in smooth muscle are mediated mainly by the PP1c and MYPT1 subunits. PP1c belongs to the phosphoprotein phosphatase (PP) family and is the main Ser-Thr phosphatase involved in several functions. The specificity of PP is achieved through its different catalytic isoforms, such as PP1, PP2, PP3, PP4, PP5, PP6, and PP7, some of which encode unique genes. PP1c is encoded by *PP1CA*, *PP1CB*, and *PP1CC*. In particular, the protein product of *PP1CB* is involved in muscle function and is controlled by various regulatory proteins, such as MYPT1, glycogen-associated regulatory subunit (GM), and myosin-binding subunit 85 kDa (MBS85), and is involved in sarcoplasmic reticulum Ca^2+^ loading and regulating several transcription factors in myocytes (Korrodi-Gregório et al., 2014; Takai et al., 2018; Lubelsky and Shaul, 2019). Various drugs and cellular regulators that inhibit PP1c are described in Table 1.

**TABLE 1:** Inhibitors of PP1c and ROCK.

Inhibitor	Effects of the inhibitor on the protein phosphatase subunit (PP1c)	References
Calyculin A (CL-A)	Potent inhibitor of PP2A and PP1 isolated from the marine sponge Discodermia calix. CL-A binds to hydrophobic, acidic and C-terminal sites of PP1c through hydrophilic, hydrophobic and metal interactions, respectively.	Murányi et al., 2005; Takai et al., 2017
Inhibitor-1	Endogenous and specific inhibitor of PP1 that increases its inhibitory capacity after phosphorylation by PKA at Thr^35^. Inhibitor-1 is heat-stable and interacts by binding to the RvXF motif and acid region of PP1c.	Lipskaia et al., 2014
A-kinase anchoring proteins (AKAP)	Multienzyme scaffolding complex that targets PKA via various substrates, including PP1. In particular, AKAP220 can anchor PP1 through a modified KVxF motif.	Schillace et al., 2001
CPI-17	Endogenous 17 kDa polypeptide that becomes an inhibitor of PP1c after being phosphorylated at Thr^38^ by ZIPK, PKC, ILK, or ROCK. Phosphorylated CPI-17 inhibits PP1c by interacting with Asp^137^, Asp^193^, Arg^220^, Tyr^271^, and Glu^274^ in the PP1c active site.	Kiss et al., 2019; Sun et al., 2019
Phosphatase holoenzyme inhibitor (PHI-1)	Member of the CPI-17 family that is an endogenous inhibitor of PP1c activated by ROCK- or ILK-mediated phosphorylation at Thr^57^. Active PHI-1 interacts directly with the active site of PP1c.	Eto and Brautigan, 2012

**Inhibitor**	**Effects of the inhibitor on ROCK**	**References**

Fasudil	Drug derived from isoquinoline that is a nonspecific ROCK1 and ROCK2 inhibitor via competitive binding at ATP binding sites in the ROCK catalytic domain. Fasudil (1-(5-isoquinolinesulfonyl)-homopiperazine) has been approved for clinical use.	Chen et al., 2013; Hamano et al., 2019
Y27632 Y39983	Cell-permeable and potent inhibitor of ROCK1 and ROCK2 via competitive binding at ATP binding sites in the ROCK catalytic domain. Potent inhibitor of ROCK1 and ROCK2 via competitive binding at ATP binding sites in the ROCK catalytic domain. Y-39983 has an inhibitory effect on ROCK 30 times greater than that of Y-27632.	Hamano et al., 2019; Shan et al., 2019
H1152	Highly specific, potent and reversible inhibitor of ROCK via competitive binding at ATP binding sites in the ROCK catalytic domain.	Hamano et al., 2019

A second pathway regulating MLCP involves phosphorylation of the phosphatase regulatory subunit MYPT1. This protein has multiple phosphorylation sites that negatively or positively regulate MLCP activity. The most important phosphorylation sites in MYPT1 are Ser and Thr residues (Chen et al., 2015). In particular, MYPT1 phosphorylation at the Ser^507^, Thr^853/850^, and Thr^696/695^ residues mediates the inhibition of MLCP activity and therefore the inhibition of airway smooth muscle relaxation. In addition, MYPT1 phosphorylation at Ser^965^, Ser^696^, and Ser^852^ is also observed in airway smooth muscle.

**FIGURE 1:**
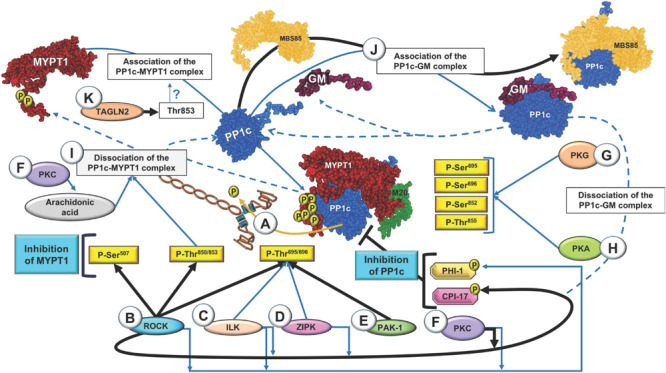
Regulation of myosin light-chain phosphatase (MLCP) through regulation of myosin phosphatase target subunit 1 (MYPT1). **(A)** Active MLCP dephosphorylates the light chain of myosin (MLC20) at the Ser^19^ residue and induces smooth muscle relaxation. **(B)** Signaling pathway mediated by RhoA kinase (ROCK). Several agonists activate the RhoA–ROCK pathway. RhoA activation is dependent on GTP and can induce ROCK activity. ROCK phosphorylates the MYPT1 subunit of MLCP to inhibit its activity and maintain smooth muscle contraction. **(C)** Signaling mediated by integrin-linked kinase (ILK). ILK phosphorylates MYPT1 at Thr^696^, inhibiting its phosphatase activity or activating the endogenous PP1c inhibitors CPI-17 and phosphatase holoenzyme inhibitor (PHI-1) by phosphorylation. **(D)** Signaling mediated by Zipper-interacting protein kinase (ZIPK). ZIPK phosphorylates MYPT1 at Thr^696^ to inactivate MLCP. **(E)** Signaling mediated by p21-activated kinase (PAK-1). **(F–H)** Activation pathway of MYPT1 mediated by protein kinases. **(F)** Protein kinase C (PKC) is involved in the activation of CPI-17, PHI-1 and arachidonic acid and dissociation of the PP1c–MYPT1 complex, which directly inactivates MLCP and results in sustained contraction. **(G,H)** Activation of cAMP-dependent protein kinase A (PKA) and cGMP-dependent protein kinase G (PKG) is responsible for phosphorylation of MYPT1 at various serine residues and one threonine residue. **(I)** Phosphorylation of the regulatory subunit MYPT1 at Thr^850^ and PKC/arachidonic acid induce dissociation of the PP1c-MYPT1 complex. **(J)** After dissociation of the complex, PP1c binds to another of its regulatory subunits, such as glycogen-targeting subunit of protein phosphatase 1 (GM) or myosin-binding subunit 85 kDa (MBS85). GM also competes with MYPT1 for binding with the PP1c catalytic subunit. PKA is involved in dissociation of the PP1c–GM complex. **(K)** Transgelin-2 (TAGLN2) dephosphorylates MYPT1 at Thr^853^. The black and blue arrows show the pathways in airway smooth muscle and in tissues other than airway smooth muscle, respectively. The blue dotted lines indicate the known pathways leading to dissociation of the PP1c–MYPT1 and PP1c–GM complexes.

Figure 1 (black arrows) shows that various kinases are involved in the phosphorylation of these sites in MYPT1 and in the regulation of PP1c in airway smooth muscle. In addition, Figure 1 also includes findings in other tissues (in blue arrows) that indicate promising future research directions in airway smooth muscle.

## Rho-Associated Kinase (ROCK) Is a Key Regulator of Airway Smooth Muscle Hypercontractility

The role of ROCK in airway smooth muscle contractility has been widely studied (Sakai et al., 2017). RhoA is a small GTPase that binds to several surface receptors that activate ROCK when it is bound to GTP (Strassheim et al., 2019). Expression of this kinase is increased in the airways of patients with asthma (Wang et al., 2020) and some drugs that inhibit ROCK signaling, such as Y27632, Y39983, H1152, and fasudil (Table 1), are highly important because they induce airway smooth muscle relaxation.

In a study with ROCK *knockout* mice in 2015, Kasahara and co-workers reported that ROCK expression is directly correlated with the increase in hyperresponsiveness caused by exposure to ozone. Similarly, they determined that reduced expression of either ROCK isoform – ROCK1 or ROCK2 – prevents the development of hyperresponsiveness. In fact, the degree of hyperresponsiveness and MLCP regulation are directly related, because the degree of hyperresponsiveness is associated with increased ROCK expression in the airway, which in turn results in an inhibitory effect via MLCP phosphorylation (Ojiaku et al., 2018; Koziol-White and Panettieri, 2019). In addition, the mechanisms by which ROCK regulates MLCP include the activation or inhibition of not only many proteins related to MYPT1 but also some that modify PP1c activity (Shahbazi et al., 2020).

### Activation of PP1c Inhibitors by ROCK

ROCK activation induces the phosphorylation of many proteins of the contractile machinery, including endogenous inhibitors of MYPT1 and PP1c (Table 1 and Figure 1) (Murányi et al., 2005). For example, in rat bronchial smooth muscle, ROCK is involved in the phosphorylation of the 17 kDa protein CPI-17 (Sakai et al., 2007). In addition, phosphorylation of CPI-17 at Thr^38^ inhibits PP1c expression and phosphatase activity (Eto et al., 1995, 1997). The CPI-17 protein is highly expressed in tonic smooth muscles such as airway smooth muscle (Takai et al., 2018). Other studies have shown that in bovine tracheal smooth muscle, the expression of neither CPI−17 nor ROCK1 was altered in MYPT1−deficient tracheal smooth muscle (Gao et al., 2017) suggesting a putative relation between these proteins.

Another inhibitor of PP1c is the phosphatase holoenzyme inhibitor (PHI-1); PP1c inhibition by PHI-1 results in ROCK-activated MLCP inhibition (Deng et al., 2002). However, the role of PHI-1 in airway smooth muscle is unknown.

### Inhibitory Phosphorylation of MYPT1 by ROCK

In a series of studies, Murányi et al. (2005) assessed several inhibitors, including Y-27632, which produced a marked decrease in MYPT1 Thr^850^ phosphorylation but had little effect on Thr^695^ phosphorylation. Another inhibitor examined was calyculin A, which acts directly on the catalytic subunit PP1c and also affects MYPT1 by increasing Thr^850^ dephosphorylation without noticeably affecting the phosphorylation level of Thr^695^. The lack of inhibitory phosphorylation at Thr^695^ may be due to phosphorylation by kinases other than ROCK, suggesting that each phosphorylation event can independently regulate MYPT1 and is mediated by different kinases. For example, p21-activated kinase (PAK-1), a protein activated by ROCK, can also induce the phosphorylation of MYPT1 at Thr^696^ in airway smooth muscle (Zhang et al., 2016, 2018; Figure 1). Another site that regulates MYPT1 is Ser^507^. Ojiaku and coworkers demonstrated that airway hyperresponsiveness and airway smooth muscle hypercontraction induced by transforming growth factor β1 in human lung slices and cells was mediated by the Smad3-ROCK pathway through MYPT1 phosphorylation at Ser^507^ (Ojiaku et al., 2018).

Although ROCK has also been shown to phosphorylate MYPT1 at both Thr^696^ and Thr^853^ *in vitro*, it acquired great relevance in models of *in vivo* contraction because ROCK is the only kinase known to phosphorylate Thr^853^. This phosphorylation event has been used as an indicator of cellular ROCK activity (Murányi et al., 2001). In cultured A7r5 rat embryonic aorta smooth muscle cells, ROCK-mediated phosphorylation of MYPT1 at Thr^695^ is similar to that of phosphorylation of human MYPT1 at Thr^696^, and phosphorylation of rat MYPT1 at Thr^850^ is similar to that of phosphorylation of human MYPT1 at Thr^853^ (Murányi et al., 2005). In a guinea pig model of asthma and in airway smooth muscle from asthma patients, increased phosphorylation of MYPT1 occurs at Thr^696^ (Álvarez-Santos et al., 2016; Yoo et al., 2017). In addition, phosphorylation of MYPT1 at Thr^696^ by ROCK in bovine airway smooth muscle inhibits MLCP activity, although Thr^853^ phosphorylation does not alter its phosphatase activity (Gao et al., 2017). In fact, in chicken gizzard smooth muscle, Thr^853^ phosphorylation induces dissociation of the PP1c-MYPT1 complex (Velasco et al., 2002) and studies have suggested that other regulatory subunits, such as MBS85 in airway smooth muscle (Gao et al., 2017) or GM in other tissues (Strålfors et al., 1985) can be associated with PP1c to maintain phosphatase activity (Figure 1).

## Evidence of Other Regulatory Units Involved in MLCP Regulation

The PP1c subunit associates with MYPT1 in addition to multiple regulatory subunits, such as GM and MBS85. This commonality arises from the structural similarity of these regulatory subunits of PP1c, mainly in the specific region of PPc1 interaction, with regions of overlap between the subunits (Korrodi-Gregório et al., 2014). In chicken gizzard smooth muscle, dissociation of the PP1c–MYPT1 complex induced by ROCK releases PP1c and allows it to associate with GM (Velasco et al., 2002; Figure 1). In the presence of dephosphorylated MYPT1, the PP1c–GM complex tends to dissociate, resulting in the interaction between PP1c and MYPT1 and subsequent dephosphorylation of MLC20 to promote smooth muscle relaxation (Johnson et al., 1996). In airway smooth muscle, transgelin-2, a small 22−kDa actin−binding protein, induces relaxation through dephosphorylation of MYPT1 at Thr^853^ (Yin et al., 2018) although whether it induces the association of the PP1c–MYPT1 complex in airway smooth muscle is unknown (Figure 1). In other tissues, the GM subunit can prevent PP1c-MYPT1 from inducing relaxation in portal vein smooth muscle, and GM slows the relaxation of the femoral artery because it competes with MYPT1 for binding with PP1c (Gailly et al., 1996). Finally, GM can be inhibited by PKA-mediated phosphorylation (Figure 1), which causes dissociation of the PP1c–GM complex (Hubbard and Cohen, 1989). During glycogen metabolism in muscles, this PKA-induced dissociation is important because the GM-PP1c complex enhances the activity of the phosphatase toward glycogen phosphorylase and glycogen synthase (Walker et al., 2000).

The MBS85 regulatory subunit is similar to MYPT1 in that both contain an RvxF motif in the N-terminus and a leucine zipper motif in the C-terminus (Grassie et al., 2011). Gao et al. (2017) found that the expression levels of MBS85 and PP1c in bovine and murine tracheal smooth muscle are higher than that of MYPT1. Additionally, these researchers did not observe changes in MLC20 phosphorylation or contraction in stimulated smooth muscle in mice with MYPT1 knockout or knock−in T853A mutation, suggesting that MLCP activity may arise from functionally shared roles of MYPT1 and MBS85. Thus, dissociation/association of MYPT1–PP1c subunits might be a mechanism underlying the regulation of MLCP activity.

## Role of Zipper-Interacting Protein Kinase (ZIPK)-Mediated Contraction

ZIPK has a zipper design similar to those of MYPT1 and M20 (Niiro and Ikebe, 2001). In human airway smooth muscle, ZIPK di-phosphorylate MLC20 at Ser^19^ and Thr^18^ to increase smooth muscle contraction. The RhoA/ROCK-inhibiting protein myosin phosphatase Rho interacting protein (p116^Rip^) was shown to induce this di-phosphorylation of MLC20 via a mechanism independent of ROCK and dependent on ZIPK (Lan et al., 2015; Komatsu et al., 2020). In colon smooth muscle, ZIPK can activate CPI-17 (Takai et al., 2018), although whether such activation occurs in airway smooth muscle is unknown. Furthermore, in an *in vitro* assay, ZIPK directly inhibited MLCP by phosphorylating MYPT1 at Thr^696^ (Murányi et al., 2002; Figure 1). The subcellular localization of the RhoA/ROCK signaling components has raised questions related to the location of ROCK in the cytoskeleton when it phosphorylates MYPT1. The existence of an intermediate binding protein between ROCK and MYPT1 has been speculated, and a ZIPK-like protein has been proposed as this link (Figure 1) because this binding is blocked when ZIPK is present (MacDonald et al., 2001).

## Role of Protein Kinase-Mediated Contraction

Smooth muscle relaxation can be generated by several mechanisms, including the formation of cyclic nucleotides such as cAMP that activate PKA or cGMP, which in turn activates PKG. MYPT1 can be phosphorylated by PKG and PKA at three serine residues – Ser^695^, Ser^697^ and Ser^852^ – and at Thr^855^ without affecting MLCP (MacDonald and Walsh, 2018; Figure 1). Additionally, *in vivo* data suggest that phosphorylation at Ser^695^ attenuates inhibitory phosphorylation of Thr^696^ in rabbit ileum smooth muscle via competition between these phosphorylation sites. The same study showed that thiophosphorylation of MYPT1 by PKA reduces phosphorylation catalyzed by ROCK, thereby preventing MLCP inhibition and inhibiting smooth muscle contraction (Wooldridge et al., 2004).

On the other hand, the effects of PKC on Ca^2+^ sensitization are increased in smooth muscle. After PKC activation, arachidonic acid enhances MLC20 phosphorylation in smooth muscle of the rabbit femoral artery due to inhibition of MLCP by dissociation of the enzymatic complex, demonstrating the participation of non-metabolized fatty acids in the regulation of smooth muscle contraction (Gong et al., 1992). Therefore, in mouse small intrapulmonary airways, PKC activation induces phosphorylation of CPI-17 (Mukherjee et al., 2013) and activation of PHI-1 by PKC has been observed in other smooth muscles (Eto and Kitazawa, 2017; Figure 1).

## Integrin-Linked Kinase (ILK) as Regulator of MLCP in Airway Smooth Muscle

Although an increase in Ca^2+^i is necessary for MLCK activation, MLC20 can be phosphorylated by ILK without an increase in Ca^2+^i (Williams et al., 1987). In addition, ILK can inhibit MLCP by phosphorylating MYPT1 at Thr^696^ or through the activation of endogenous inhibitors of PP1c, such as CPI-17 and PHI-1 (Deng et al., 2002; Table 1 and Figure 1). Another study determined that ILK, which is generally localized to the plasma membrane, was also localized on cytoskeletal myofilaments (Deng et al., 2001). The myofilament-localized ILK isoform contains a series of ankyrin regions similar to those in the PP1c subunit of MLCP, and this isoform may interact with MLC20 (Lynch et al., 1999). However, its role in hypercontractility and hyperresponsiveness is unclear, because ILK expression has been demonstrated to be reduced in experimental asthma (Álvarez-Santos et al., 2016).

In conclusion, airway smooth muscle hypercontractility might result from inhibition of PP1c, inhibitory phosphorylation of MYPT1 and/or dissociation of the PP1c/MYPT1 complex. Many signaling pathways converge in the regulation of MLCP, a key regulator of contraction; however, other potential perspectives on the hypercontractile phenotype of airway smooth muscle might remain to be explored.

## Author Contributions

MÁ-S and BB-P contributed to the conception and design of the work. MÁ-S and MÁ-G wrote the first draft of the manuscript. BB-P and SE-S revisited critically the intellectual content. All authors contributed to the manuscript revision, read and approved the submitted version.

## Conflict of Interest

The authors declare that the research was conducted in the absence of any commercial or financial relationships that could be construed as a potential conflict of interest.
